# Natural Allelic Variation Defines a Role for *ATMYC1*: Trichome Cell Fate Determination

**DOI:** 10.1371/journal.pgen.1002069

**Published:** 2011-06-09

**Authors:** V. Vaughan Symonds, Greg Hatlestad, Alan M. Lloyd

**Affiliations:** Institute for Cellular and Molecular Biology, The University of Texas at Austin, Austin, Texas, United States of America; Peking University, China

## Abstract

The molecular nature of biological variation is not well understood. Indeed, many questions persist regarding the types of molecular changes and the classes of genes that underlie morphological variation within and among species. Here we have taken a candidate gene approach based on previous mapping results to identify the gene and ultimately a polymorphism that underlies a trichome density QTL in *Arabidopsis thaliana*. Our results show that natural allelic variation in the transcription factor *ATMYC1* alters trichome density in *A. thaliana*; this is the first reported function for *ATMYC1*. Using site-directed mutagenesis and yeast two-hybrid experiments, we demonstrate that a single amino acid replacement in *ATMYC1*, discovered in four ecotypes, eliminates known protein–protein interactions in the trichome initiation pathway. Additionally, in a broad screen for molecular variation at *ATMYC1*, including 72 *A. thaliana* ecotypes, a high-frequency block of variation was detected that results in >10% amino acid replacement within one of the eight exons of the gene. This sequence variation harbors a strong signal of divergent selection but has no measurable effect on trichome density. Homologs of *ATMYC1* are pleiotropic, however, so this block of variation may be the result of natural selection having acted on another trait, while maintaining the trichome density role of the gene. These results show that *ATMYC1* is an important source of variation for epidermal traits in *A. thaliana* and indicate that the transcription factors that make up the TTG1 genetic pathway generally may be important sources of epidermal variation in plants.

## Author Summary

Among the goals of modern evolutionary biology is to identify the molecular genetic sources of natural variation. Although genetic mapping has led to an increased understanding of the genetic architecture of natural variation, there are surprisingly few cases where the molecular source of the variation has been identified. Here, we utilize previous mapping results to identify the gene and ultimately a polymorphism that underlies natural variation for a dynamic trait in *Arabidopsis thaliana*: trichome density. We show that plants carrying a knock-out of the bHLH transcription factor *ATMYC1* have a reduced trichome density phenotype; this is the first reported function for *ATMYC1*. Using traditional and molecular genetic approaches, we identify a single base change in natural alleles of *ATMYC1*, which leads to an amino acid replacement that qualitatively alters protein–protein interactions with known partners, presumably altering the trichome cell fate pathway. In a broad screen for molecular variation in *ATMYC1*, we identify a dense block of amino acid replacements that differentiates two high-frequency allele types. Although this block of variation does not appear to affect trichome density, based on paralogs of *ATMYC1*, we propose that this variation has arisen due to directional selection on another epidermal trait.

## Introduction

Understanding the origins, maintenance, and loss of natural variation remain important goals of evolutionary biology; ideally, we should like to know what types of molecular genetic changes generate the variation that natural selection acts on. For most traits, variation is distributed continuously in natural populations, a product of polymorphisms at many loci, environmental effects, and genotype by environment interactions [1], [2]. Common first approaches to characterizing the genetic bases of natural variation include quantitative trait locus (QTL) mapping (see reviews [3]–[5]) and, more recently, genome-wide association mapping (e.g., [6]–[8]). While these methods provide many genetic insights, mapping results largely remain hypotheses regarding the molecular nature of biological diversity. To identify the genes and ultimately the polymorphisms that underlie natural variation still require detailed gene-by-gene analysis [9].

Information about the molecular changes that underlie natural variation within and among species provides important insights into the mechanisms that drive local adaptation, morphological evolution, and speciation. For example, molecular data have revealed a good deal about the evolution of flowering time in *Arabidopsis thaliana* [10]–[13], morphology in various groups [14]–[16], and speciation in *Drosophila* [17], [18]. Despite progress for specific traits, few general patterns have emerged regarding the molecular bases of natural variation. For example, King and Wilson [19] proposed the concept of “evolution at two levels” more than three decades ago, yet we still know little about the relative roles of coding versus regulatory mutations in evolution [20]. Such patterns may ultimately prove difficult to identify because they vary according to the taxonomic level of comparison, nature of the trait, or life history, but more data are required.

For plants, there are only ∼100 cases where the gene underlying natural variation has been identified and fewer than that for the causal polymorphism (reviewed in [21]). Perhaps further complicating the search for natural molecular evolutionary patterns, these data are heavily biased toward crops; however, roughly a third of the data are reported from work on the model flowering plant *Arabidopsis thaliana*. Like many model species, *A. thaliana* has a high degree of intra-specific phenotypic variation (reviewed in [3]) and a substantial functional genetic infrastructure [22], which make it an ideal system for pursuing the genes that underlie natural variation [23]. Indeed, studying highly variable traits with well-described molecular genetic underpinnings may represent our best opportunities to identify genes of interest and ultimately elucidate broad molecular evolutionary patterns. Epidermal cell fate in *Arabidopsis thaliana* represents one such system.

The interaction between an organism and its environment plays a critical role in the evolution of morphology and local adaptation [24], [25]. For individual plants, which cannot migrate away from sub-optimum conditions, this interaction is all the more important and is mediated by organs such as stomata [26], [27], root hairs [28], [29], trichomes [30]–[32], anthocyanin producing cells [33], [34], and seed coats [35]. Collectively these organs make up the plant epidermis, an elaborate skin that serves as the interface between the organism and its environment. In *A. thaliana*, epidermal cell fate is largely regulated by the TTG1 genetic pathway [36], which is mainly comprised of many pleiotropic and epistatic transcription factors and the scaffold protein, TTG1. Among the epidermal traits regulated by this pathway, trichome density is known to play a dynamic defensive role in *A. thaliana* [32]; increased trichome density under herbivorous conditions results in a fitness advantage, but individuals with higher trichome density in the absence of herbivorous insects have been shown to incur a fitness cost. While this suggests that environmental heterogeneity may maintain genetic variation for trichome density (within or between populations), we know little about the molecular nature of this variation. To date, only one QTL for trichome density has been identified [37]; *ETC2* encodes a single repeat MYB protein known to be a repressor of the trichome cell fate. This leaves the molecular nature of most trichome density variation within *A. thaliana* unexplained.

**Figure 1:**
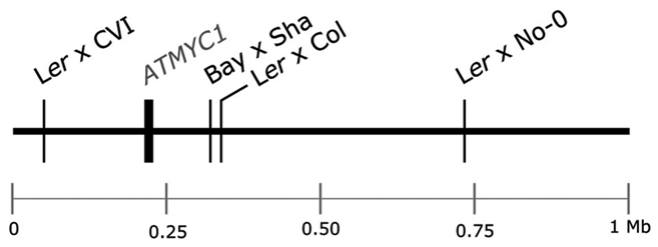
Schematic of the top 1 Mb of the physical map of chromosome four of *A. thaliana*. The physical position of the *ATMYC1* locus and the estimated physical positions of TDL5 from four different mapping populations are indicated to scale. Confidence intervals for the QTL mapped in each population span the entire top 1 Mb of chromosome four, including the *ATMYC1* locus.

Previous QTL mapping results for trichome number [38], [39] and trichome density [40] have identified multiple QTL in *A. thaliana*. One QTL mapped by Symonds *et al.* [40], TDL5, was localized to the top of chromosome four independently in each of four Recombinant Inbred Line (RIL) populations (Figure 1). Estimates of the physical position of this QTL and the similar magnitude of effect for TDL5 across mapping populations suggested that the same locus was mapped independently in each population. In an initial screen of the region, no gene with a known trichome phenotype was discovered; however, the search did reveal a bHLH transcription factor, *ATMYC1*, three paralogs of which [41], [42] have reduced trichome density phenotypes when knocked out [43]–[45]. *ATMYC1* is expressed in both leaves and seeds [46] but over-expression of the gene has yielded no observable phenotype [44]. More recently, Zimmerman *et al.* [47] demonstrated protein-protein interactions between ATMYC1 and several R2R3 MYBs with known roles in epidermal cell fate. Here, we present genetic, molecular, and protein-protein interaction data that demonstrate that *ATMYC1* is involved in epidermal cell fate and is a Quantitative Trait Gene (QTG) that underlies natural variation for trichome density. The results further reveal a complex pattern of protein evolution at *ATMYC1* with as yet undetermined origin and effects.

## Results

### *atmyc1* trichome phenotype

**Figure 2:**
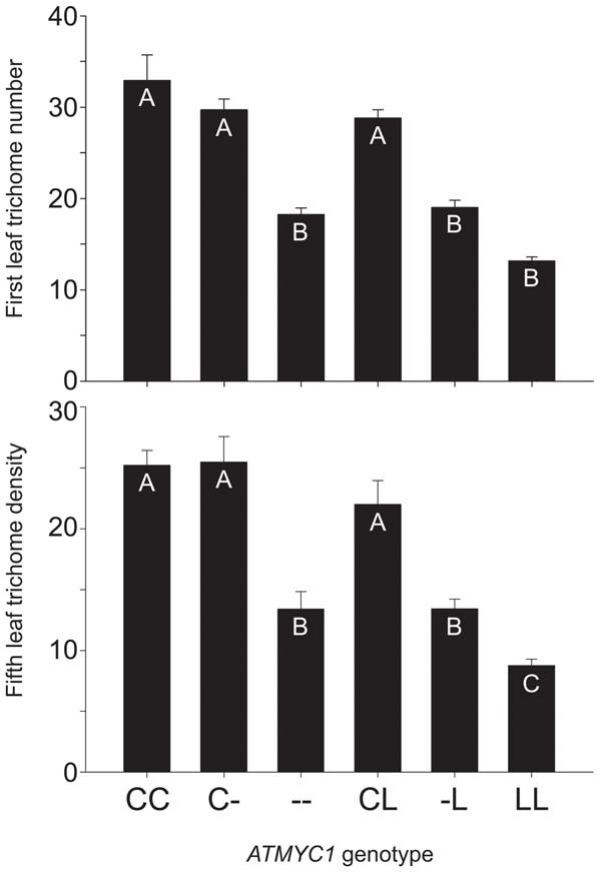
Genetic complementation test results. To test for genetic complementation of the *atmyc1* mutant, a series of test-crosses were made between Col-0, L*er*-0, and the *atmyc1* mutant (in the Col-0 background). Two phenotypes were scored for each genotype: trichome number on first true leaves and trichome density on fifth true leaves; means (+SE) are shown for each *ATMYC1* genotype and genotypes with different letters within each bar chart are significantly different from each other (*p*<0.01, except B vs. C (*p*<0.05). Both first and fifth leaf phenotypes were used in previous QTL mapping studies. Note that the trends for the two phenotypes are nearly identical, simply shifted up or down, depending on the leaf scored. By comparing the Col (CC), Col x *atmyc1* (C-) and *atmyc1* (--) genotypes (- denotes a nonfunctional mutant allele), all of which are in an otherwise Col-0 background, it is clear that a single copy of the Col allele of *ATMYC1* recovers the mutant phenotype to near wildtype levels. In contrast, comparisons between the Col x L*er* (CL) and *atmyc1* x L*er* (L-) genotypes, each of which is in an otherwise Col/L*er* background show that the L*er* allele of *ATMYC1* does not recover the mutant phenotype.

Previous QTL mapping results for trichome density in *A. thaliana* localized a QTL to the top of chromosome four in four independent mapping populations [40]. Although no known trichome regulator was apparent in this region, *ATMYC1*, a paralog of three genes with known roles in trichome initiation was discovered. To determine if *ATMYC1* has a role in trichome initiation, we examined TDNA insertion (knock-out) lines. A homozygous TDNA insertion line for *ATMYC1* (SALK_057388) in a Col-0 background was determined to have a significantly different number of trichomes/first true leaf and trichome density phenotype on fifth true leaves relative to the wildtype Col-0 accession (Figure 2). The *atmyc1* mutant produced fewer trichomes than wildtype on first true leaves and had a lower trichome density on fifth leaves. The trichome phenotype of *atmyc1* has since been verified in two additional independent TDNA insertion lines of the gene (Figure S1).

### Quantitative complementation tests for *ATMYC1*

To test for a functional difference between the Col-0 and L*er*-0 (hereafter, Col and L*er*) alleles of *ATMYC1*, quantitative genetic complementation analyses were performed by comparing the trichome densities of Col, L*er*, a homozygous *atmyc1* mutant in a Col background, and pairwise F_1_s among them (Figure 2). Germination rates were variable across genotypes in each experiment, resulting in sample sizes ranging from 11–18 and 8–14 for first and fifth leaf phenotypes, respectively. A one-way ANOVA revealed that both traits were found to differ significantly across the compared groups (first leaf phenotype: *F* (5, 81) = 42.455, *p*<0.001; fifth leaf phenotype: *F* (5, 56) = 20.63, *p*<0.001) and Tukey-Kramer post-hoc tests, which account for sample size variation, were revealing in several ways. The test cross of Col x *atmyc1* showed little to no evidence of a gene dose effect (Figure 2). That is, the Col x *atmyc1* genotype does not differ significantly from that of the Col wildtype genotype for first and fifth leaf trichome phenotypes, showing that a single Col allele of *ATMYC1* is sufficient to complement the reduced trichome phenotype of the mutant to near wildtype levels. In contrast, the Col x L*er* genotype has trichome phenotypes significantly higher than the *atmyc1* x L*er* genotype, showing that a single copy of the L*er ATMYC1* allele does not complement the *atmyc1* mutant phenotype, indicating that L*er* contains a nonfunctional (with regard to trichome initiation), recessive allele of *ATMYC1*.

### Network analysis of *ATMYC1* alleles

**Figure 3:**
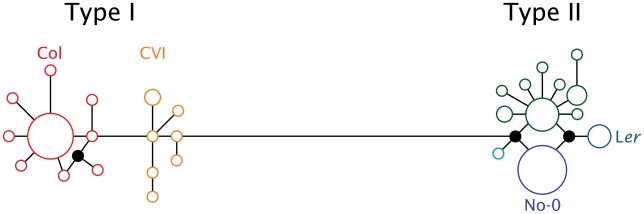
Median joining network of 28 *ATMYC1* inferred cDNA haplotypes representing alleles sampled from 72 *A. thaliana* accessions. Each circle represents a unique coding haplotype and the area of each circle indicates the relative frequency at which each haplotype was sampled. Unsampled haplotypes required to complete the network are drawn as filled-in circles. Branch lengths reflect nucleotide change between any two haplotypes. The large block of variation within exon six falls along the long branch that connects the Type I to the Type II haplotype clusters. The accessions discussed in the text are indicated adjacent to the haplotype they possess.

In a screen of 72 *A. thaliana* accessions, considerable sequence variation was discovered among natural alleles of *ATMYC1* with a total of 28 (inferred) cDNA haplotypes discovered (GenBank accession #s: JF801957-JF802028). Median-joining analyses yielded a network that is split into two diverged clusters (Figure 3); these have been labeled as Type I and Type II, with 16 and 12 haplotypes, respectively. Alleles of these two Types consistently differ by 25 substitutions, which translate to 17 amino acid replacements. Interestingly, nearly all of this variation (24 of 25 substitutions and all 17 replacements) is in exon six (Figure S2).

Both allele types are at high frequency. Of the 72 accessions for which full-length *ATMYC1* sequence was obtained, 31 possess a Type I allele and 41 have a Type II allele; however, no obvious geographic pattern was evident. With regard to the four RIL mapping populations in which TDL5 was mapped previously [40], it is interesting that the six parental accessions possess five different alleles (Figure 3; only the allele carried by the four parents of the mapping populations that include L*er* as a parent are labeled). Perhaps most interesting among these alleles is that which the L*er* accession carries, as this allele consistently conferred lower trichome density in previous QTL mapping experiments. Three natural accessions possess this same allele, one of which is La-0 (cs6765), a wildtype accession from the same region as the progenitor of L*er*; the other two are Dra-1 (cs6686) and Sg-2 (cs6859).

### Molecular evolution of the *ATMYC1* locus

**Figure 4:**
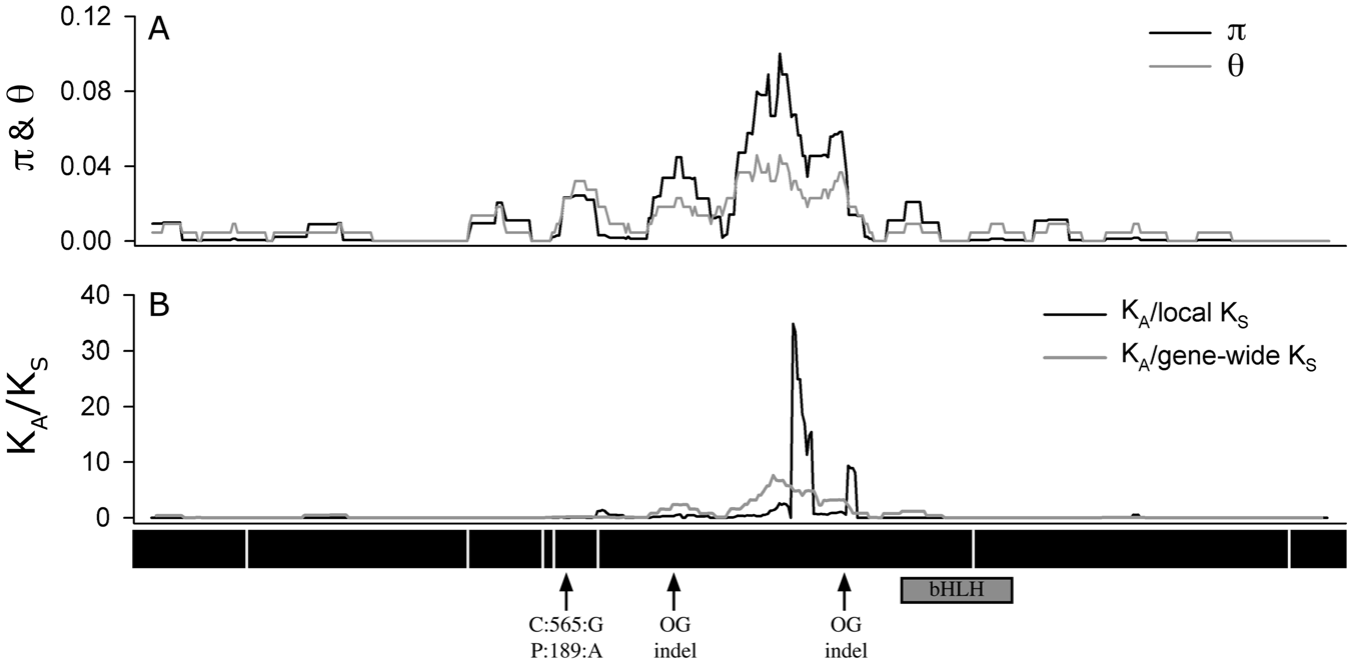
Sliding window analysis. Of (A) π and θ_W_ and (B) the ratio of nonsysnonymous substitution rate (*K*_A_) to synonymous substitution rate (*K*_S_). The diversity plots in (A) were generated with a 45 bp window moved along the length of the aligned sequences at 3 bp intervals; the black line shows π and the grey line shows θ_W_. The plots in (B) were generated by comparing the two pools of *ATMYC1* alleles (Type I and Type II) with a window size of 45 bp again slid at 3 bp intervals. The black line represents the ratio of local *K*_A_/local *K*_S_ and the grey line represents the ratio of local *K*_A_/gene-wide average *K*_S_. Values near one are inferred to represent regions having undergone neutral molecular evolution, values <<1 indicate purifying selection and values >>1 indicate positive selection. The black boxes beneath the plot represent the eight exons of *ATMCY1*. While purifying selection is evident throughout most of the gene, strong evidence for divergent selection between Types I and II alleles is present in exon six. The positions of two indels relative to outgroup (OG) taxa are indicated with arrows. Also the position of the substitution that results in an amino acid polymorphism at position 189 between Col and L*er* is indicated with an arrow. The position of the bHLH domain of *ATMYC1* is represented by a grey box.

An analysis of the 72 *A. thaliana* alleles of *ATMYC1* yielded overall levels of nucleotide diversity and polymorphism (π & θ_w_; Table 1) that are somewhat higher than genome-wide average values reported for *A. thaliana* genes [48], [49]. A sliding window analysis revealed high localized levels of nucleotide diversity (Figure 4), the highest of which was detected within exon six.

**Table 1:** Summary of diversity indices for *ATMYC1.*

Sample	π	θ_w_
Type I	0.002	0.004
Type II	0.001	0.002
Total	0.011	0.008

Because regions of high nucleotide diversity corresponded with divergence between Type I and Type II alleles, we wanted to characterize the nature of this molecular variation. To explore this, we used a sliding window method to study rates of non-synonymous (*K*_A_) and synonymous (*K*_S_) divergence between Types I and II alleles across the entire 1.58 kb coding region. These analyses revealed evidence of alternative forms of selection that are gene region-specific (Figure 4). Across most of the gene, it appears that purifying selection has acted to constrain the amino acid sequence (ratios <<1); however within exon six, extremely high rates of amino acid replacement are evident between the two Types. As a *K*_A_/*K*_S_ ratio greater than one is often cited as a conservative cut-off for positive selection [50], [51], values approaching 30 are exceptional. Even the more rigorous approach using the gene-wide average *K*_S_ value resulted in a *K*_A_/*K*_S_ ratio greater than eight in this region. Outside of exon six, no other region of *ATMYC1* showed evidence of positive selection. Interestingly, most of the divergence between the two *A. thaliana ATMYC1* Types falls between two indels that differentiate all *A. thaliana* alleles from two distant outgroup alleles (Figure 4 and Figure S2).

### Testing for an *ATMYC1* Types difference for trichome density

Of the 93 ecotypes that were screened for trichome density, 50 possessed a Type I allele and 43 possessed a Type II allele. Although broad-sense heritability was relatively high for the experiment (H^2^ = 0.71), there was no significant difference for trichome density detected between ecotypes carrying the two alternative *ATMYC1* allele Types according to a Kruskal-Wallis test (data not shown). Although variation segregating at other loci may overwhelm the effects of alternative *ATMYC1* Types, it appears that the observed sequence variation in exon six has little to no effect on trichome density. Given the sample sizes and standard deviations, a power analysis indicated that a trichome density difference of at least three units should have been detectable as significant.

### Conservation at Col/L*er* polymorphism positions

Because a QTL was mapped for trichome density near *ATMYC1* in the Col x L*er* RIL population and quantitative genetic complementation tests revealed a functional difference between the Col and L*er ATMYC1* alleles, we examined polymorphisms between these two alleles. The Col and L*er* accessions possess different *ATMYC1* Types; however, the variation in exon six that distinguishes the two Types has no detectable effect on trichome density. Therefore, other polymorphisms between the Col and L*er* alleles were investigated. The Col and L*er* proteins differ at just four other positions: A13T, E83Q, P189A, and P343H (Col:aa position:L*er*). Of these polymorphisms, only position 189 is highly conserved across proteins and taxa. Out of 100 homologs, representing monocots and dicots, retrieved through a protein-BLAST search of the Col ATMYC1 protein sequence, all 100 shared the Col state (proline) at ATMYC1 amino acid position 189 (data not shown). This position is also of interest as it resides within an undefined, but known MYB interaction domain in the amino end of close paralogs of *ATMYC1* [44], [45]. The other three polymorphic positions were found to be far less conserved. Based on these results, yeast-2-hybrid experiments focused on the highly conserved position 189 and the non-conserved position 13 as a control.

### Yeast two-hybrid results

**Figure 5:**
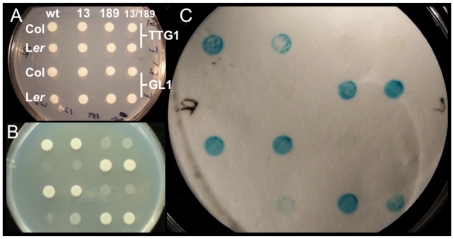
Yeast two-hybrid analysis testing Col/L*er* ATMYC1 polymorphism effects on protein–protein interactions with known binding partners TTG1 and GL1. All images show the same pattern of yeast containing ATMYC1 from Col or L*er* in binding domain with either TTG1 or GL1 in the activation domain. To test whether variation at amino acid positions 13 or 189 had any effect on the function of ATMYC1, reciprocal replacements were produced between Col and L*er* at these positions separately and together; wt shows interaction data for the native Col and L*er* ATMYC1 proteins, 13 shows interaction data for reciprocal replacements in Col and L*er* alleles at amino acid position 13, 189 shows interaction data for reciprocal replacements at position 189, and 13/189 shows interaction data for alterations at both positions. (A) Control colonies with no selection for interaction. (B) Same as A grown on Histidine dropout media. Interaction between ATMYC1 and either TTG1 or GL1 is required for growth. (C) Colonies in A were lifted on nitrocellulose, which was used in analysis of β-galactosidase activity. Blue is an indication of interaction. These results clearly show that the Col ATMYC1 allele interacts with GL1 and TTG1 while the L*er* allele does not. Reciprocal replacements clearly show that the cause of the interaction difference between Col and L*er* proteins is the polymorphism at amino acid position 189; the Col protein with 189A no longer interacts with TTG1 and GL1 and the L*er* protein with 189P fully interacts with TTG1 and GL1. Reciprocal replacements at position 13 have no effect on binding and the combined replacements at 13/189 mirror the results of the replacement at position 189 alone.

We investigated the effects of two Col/L*er ATMYC1* polymorphisms on protein-protein interactions using binding assays with known partners, TTG1 and GL1 [47], [52]. The results are clear. ATMYC1 encoded by the native Col allele interacts with TTG1 and GL1 and the ATMYC1 protein encoded by the native L*er* allele does not. Reciprocal replacements at position 13 for the Col and L*er* alleles had no effect on binding, while reciprocal replacements at position 189 qualitatively altered binding for both proteins. Specifically, when the Col allele was changed to match the L*er* allele at position 189, the protein no longer bound to TTG1 or GL1 and when the L*er* allele was changed to match the Col allele at position 189, the resulting protein then bound with TTG1 and GL1 (Figure 5).

## Discussion

Identifying the genes and ultimately the molecular changes that underlie natural morphological variation remain important, but largely elusive goals. Here we have taken a candidate gene approach based on QTL mapping results and have identified a function for the transcription factor, *ATMYC1*, in which natural molecular variation affects trichome density in *A. thaliana*. Furthermore, our results reveal a strong signal for directional selection restricted to one exon of *ATMYC1* that has no detectable effect on trichome density but may suggest a pleiotropic role for the gene.

### *ATMYC1* is a Quantitative Trait Gene for trichome density

An initial TDNA insertion line of *ATMYC1* in the Col background was found to have a reduced trichome density phenotype relative to wildtype Col (Figure 2); subsequent examination of additional independent insertion lines have confirmed this trichome initiation phenotype (Figure S1). The trichome phenotype is consistent with high sequence similarity between *ATMYC1* and close paralogs *GL3, EGL3*, and *TT8* [41], [42], which also have trichome phenotypes upon knock-out [43]–[45]. However, this finding is somewhat surprising, as *GL3* and *EGL3* have been shown to be sufficient to fulfill the bHLH role in trichome initiation; a *gl3*/*egl3* double mutant is completely glabrous [45], suggesting that *ATMYC1* may be an enhancer of *GL3* and *EGL3*. While the precise genetic role that *ATMYC1* plays in the TTG1 pathway requires more work to elucidate, the trichome cell fate function is clear. Subsequent quantitative genetic complementation tests showed that the L*er* allele of *ATMYC1* cannot recover the *atmyc1* trichome phenotype, while the Col allele recovers it completely (Figure 2), indicating that natural molecular variation in *ATMYC1* alters trichome density.

Sequence variation between Col and L*er* (parents of the one of the mapping populations where TDL5 was mapped) *ATMYC1* alleles prompted a broad survey of *ATMYC1* from 69 ecotypes and three lab strains of *A. thaliana*, which revealed a striking pattern of high frequency polymorphism. The coding region of the two primary Types observed consistently differ by 25 substitutions, which translate into 17 amino acid replacements; 24 of the 25 substitutions and all 17 replacements reside within exon six. This amounts to >10% amino acid replacement within exon six; no other region of the gene has a high rate of replacement. Allelic dimorphism has been reported for some, but not all, other loci in *A. thaliana* (e.g., [37], [53]–[55]) and is likely the result of diverfgence between two long-isolated populations of *A. thaliana* with subsequent break-down of population subdivision and admixture. Regardless of the origin of the Types, the *ATMYC1* results are striking due to the high frequency of amino acid replacement and because nearly all of the variation resides within one relatively small region of the gene. That the Col and L*er* alleles possess different *ATMYC1* Types initially suggested that the molecular variation in exon six might explain the functional difference revealed by quantitative complementation tests; however, neither association tests nor yeast 2-hybrid experiments (Figure 5) support this hypothesis.

Outside of the block of variation in exon six that differentiates the two Types, only four other changes differentiate the coding regions of the Col and L*er* alleles. Yeast-2-hybrid experiments to test the known interactions between ATMYC1 and TTG1 and ATMYC1 and GL1 showed that the P189A (Col:aa position:L*er*) replacement has a qualitative effect, completely eliminating these interactions for the protein encoded by the L*er* allele of *ATMYC1*. The proline at this position is conserved, even among distant homologs of *ATMYC1*, and likely resides in a known, but undefined, protein-protein interaction domain identified in close paralogs of *ATMYC1* [44], [45]. Indeed, simply replacing the proline for an alanine at this position in the Col allele eliminates the interaction with TTG1and GL1, while the reciprocal change, replacing the alanine for a proline in the L*er* allele, restores these interactions (Figure 5). Although this may not have been the first or the only mutation in the P189A *ATMYC1* allele to eliminate gene function and reduce trichome density, these data show that the P189A replacement is sufficient to explain the functional difference between the Col and L*er* alleles, presumably by altering trichome initiation, thereby decreasing trichome density.

We conclude that *ATMYC1* is a Quantitative Trait Gene (QTG) for trichome density in *A. thaliana* and that the mutation at DNA position 565 is a Quantitative Trait Nucleotide (QTN) for the trait. An interesting point to emerge here is that a single base substitution has lead to a qualitative breakdown in protein-protein interaction, which has a quantitative phenotypic effect; based on sequence similarity, this is likely due to functional redundancy between *ATMYC1*, *GL3*, *EGL3,* and *TT8*. The nature of the L*er* mutation suggests that this was the same QTG mapped for trichome density by Symonds *et al.* [40] in two other populations that have L*er* as a parent: No-0 x L*er* and CVI x L*er*; CVI and No-0 possess Type I and II *ATMYC1* alleles, respectively, but share the proline at amino acid position 189 with Col, further supporting the conclusion that the variation differentiating the Types has little or no effect on trichome density while the replacement at position 189 underlies the mapped effect. Furthermore, QTL mapping results show that the L*er* allele at TDL5 consistently confers lower trichome density than the alternative allele from Col, CVI, and No-0 and the additive effect of TDL5 was nearly identical in all three mapping populations [40].

The *ATMYC1* allele carried by L*er* is shared by three natural ecotypes, La-0 (Poland), Dra-1 (Czech Republic), and Sg-2 (Germany). In our sample, this allele is at a frequency of ∼5%. Because it is unknown if *ATMYC1* is pleiotropic, we cannot yet address whether or not the replacement at position 189 affects other traits. However, the 189A allele shows no signs of degradation to pseudogene status in any of the three natural ecotypes. This could be due to one or both of the following: (1) the protein has other functions that are not affected by the mutation at position 189 and is maintained by purifying selection and (2) this mutation is relatively recent and there has not been sufficient time for other mutations to accumulate. With regard to the second hypothesis, the 189A allele has at least persisted long enough for migration to increase its presence to multiple populations.

### Cryptic allelic variation at *ATMYC1*

Association tests showed no obvious trichome density difference for the two high frequency *ATMYC1* Types and yeast-2-hybrid experiments suggest that the divergence between the two Types has no effect on known protein-protein interactions. If the variation in exon six has no effect on trichome density, then what explains the clear signature of divergent selection between the allele Types? There would seem to be three logical explanations. First, the association test results could reflect confounding factors, such as segregation of variation at other loci that have larger effects on trichome density and essentially swamp out a potential *ATMYC1* Types effect. If this were true, the Types effect would have to be in addition to and much weaker than that found for the replacement at position 189.

Second, divergence could have been in response to selection on a trait other than trichome density; indeed, paralogs of *ATMYC1* (*GL3*, *EGL3* and *TT8*) are all pleiotropic for several epidermal traits [43]–[45], *ATMYC1* has been shown to interact with several MYB transcription factors that coordinate other epidermal fates [47], and an *ATMYC1* homolog from *Vitis vinifera* (Vitaceae) was recently shown to have an epidermal (anthocyanin) phenotype [56]. *ATMYC1* is most highly expressed in “seeds” [46], therefore, it may be expected to be involved in testa development as well; however, we have observed no differences in comparisons between a TDNA knock-out line of *ATMYC1* in the Col-0 background and Col-0 for three seed coat traits: mucilage production, condensed tannin synthesis, and seed coat cell morphology (data not shown).

Finally, the two Types may have evolved independently in response to deleterious indels. Comparisons with outgroup homologs of *ATMYC1* show that the divergence between the two *A. thaliana* allele Types resides between or near two indels (relative to outgroup taxa) of 18 and 15 bp after coding DNA positions 705 and 927 (in Col-0 sequence), respectively (Figure 4 and Figure S2; outgroup sequence data not shown). Specifically, rather than diverging from one another, the two *A. thaliana* Types may have independently diverged away from a common nonfunctional ancestral copy of the gene. Although at this stage we cannot determine the origins of the indels, recombination and transposable elements seem likely candidates. Whatever the origins, in *A. thaliana*, isolated populations may have acquired independent compensatory mutations that became fixed in each lineage. Because trichome density is dynamic, with the fitness of a given density being relative to the environment [32], such mutations may persist for long periods, thus allowing time for compensation. All *A. thaliana* alleles share indel states at these two positions with *A. lyrata* relative to the more distant outgroups, *Capsella bursa-pastoris* and *Crucihimalaya himalaica*. Further functional and analytical tests will be required to resolve the origins and potential effects of the divergence around these indels.

### Conclusions

Trichome density in *A. thaliana* is likely to be under alternating forms of selection, depending on the particular environment in which a plant resides. The TTG1 genetic pathway, which contains multiple and various types of transcription factors, many of which are functionally redundant, would seem a likely reservoir of genetic variation for epidermal traits and a prime pathway for “genetic tinkering” [57] with potentially a low risk of permanent unidirectional trait change. Indeed, we have shown here that a low frequency polymorphism that results in a simple amino acid replacement in *ATMYC1* reduces trichome density in natural ecotypes of *A. thaliana*, thereby ascribing the first function to *ATMYC1*. Our results also revealed a high frequency block of amino acid replacements in *ATMYC1* with as yet unknown effects. *ATMYC1* is the second gene in the TTG1 pathway recently identified to affect natural quantitative variation for trichome density; interestingly, for the single-repeat MYB, *ETC2*, high frequency polymorphisms do affect trichome density [37], while a similar pattern in *ATMYC1* does not seem to alter trichome density. Clearly patterns that define the types of mutations and classes of genes that underlie natural variation may be difficult to identify; however, the TTG1 pathway is quickly emerging as a good place to search.

## Methods

### The *ATMYC1* mutant phenotype

A TDNA insertion line (SALK_057388) for the *ATMYC1* locus (At4g00480) in the Col-0 background was obtained from The *Arabidopsis* Biological Resource Center (ABRC; http://signal.salk.edu/cgi-bin/tdnaexpress). The initial batch of seed was screened using a standard PCR protocol to identify a lineage homozygous for the TDNA insertion, which resides in the first exon of the gene. Based on initial observations of a trichome density phenotype for the *atmyc1* mutant, first leaf trichome number and fifth leaf trichome density phenotypes were then measured in replicates of Col-0 and *atmyc1*as described in the following section.

### Quantitative genetic complementation tests

To test the hypothesis that variation at the *ATMYC1* locus underlies trichome density variation mapped to TDL5 in previous QTL studies [40], quantitative complementation tests were performed. In the QTL mapping studies, the L*er* allele at TDL5 was shown to confer lower trichome density than the alternative parents' (Col-4, CVI, and No-0) alleles in each mapping population. However, because the available *atmyc1* knock-out mutation is in the Col-0 background, the most direct comparison that could be made (with regard to QTL mapping results) was between Col and L*er*. Crosses were made by transferring pollen from flowers of the L*er* accession onto the stigmatic surface of emasculated flowers of the *atmyc1* mutant and of Col wildtype. To control for potential cytoplasmic variation among accessions, all crosses were made with Col or *atmyc1* as the pollen recipient. Therefore, the resulting F_1_s differ only at the *atmyc1* locus. This allowed for comparisons between individuals with a Col/L*er* and an *atmyc1*/L*er* genotype at *ATMYC1*, while holding the rest of the genome constant. That is, the only difference between the two sets of progeny is the replacement of a copy of the Col allele with a null (mutant) *atmyc1* allele. To test for a dosage effect, Col was crossed to *atmyc1*, which yields a Col individual with a single functional *ATMYC1* allele (*atmyc1*/Col genotype). Twenty replicates of each F_1_ genotype, parental accession, and the *atmyc1* mutant were potted and the pots were randomized across four flats. All seed were vernalized in the dark for four days at 4°C, and subsequently moved to a fluorescently lit 20°C growth chamber. Sixteen days after emergence the number of trichomes on each of the first two true leaves of each seedling were counted under 50X magnification on a dissecting microscope; this is referred to as the “first leaf” trichome number phenotype. For the “fifth leaf' trichome density phenotype, the same experiment was set up as described above and trichome density was measured on the fifth true leaf at 21 days after emergence, as described by Symonds *et al.* [40]. The mean for each trait was then calculated from these data for each genotype. The genetic contribution to trichome number and trichome density variation was evaluated for first and fifth leaf phenotypes independently by ANOVA and unplanned pairwise comparisons between genotypes following the Tukey-Kramer method as described by Sokal and Rohlf [58].

### Survey of natural allelic variation at *ATMYC1*

DNAs were isolated from 69 natural accessions and three lab strains of *A. thaliana* acquired from the ABRC (Table S1), following a modified CTAB method [59]. Primers were designed from the Col-0 *ATMYC1* sequence (GenBank accession #NC003075) to PCR amplify the open reading frame plus ∼200 bp up- and down-stream of the start and stop codons, respectively. Primers corresponding with the first and last 21 bp of the Col-0 *ATMYC1* cDNA sequence were used to amplify the *ATMYC1* homolog from outgroup taxa (all Brassicaceae): *Arabidopsis lyrata*, *Crucihimalaya himalayica*, and *Capsella bursa-pastoris*. All primers were used with manufacturer-supplied 1X *Taq* buffer, 1U AccuPrime High-Fidelity *Taq* polymerase (Invitrogen Inc.), and ∼20 ng genomic DNA in 20 uL reactions. PCR samples were checked for amplification success on 0.7% agarose gels stained with ethidium bromide, and were subsequently purified in Multiscreen PCR clean-up plates (#MANU03050, Millipore). Approximately 100 ng of each purified PCR product were then used in each of seven sequencing reactions using primers designed to anneal at staggered internal positions, providing a minimum of two overlapping sequences across the entire gene. Allelic contigs were constructed for each ecotype and sequence editing and validation were performed using sequencher v.4.2.2 (Gene Codes Corp.). Full-length genomic sequences of *ATMYC1* for all accessions were aligned initially using clustalx v.1.83 [60], and subsequently corrected by hand. To generate inferred cDNA sequence alignments, introns were identified using the published Col-0 cDNA sequence as template (Arabidopsis Genome Initiative) and non-coding DNA sequence was removed from the genomic alignment in bioedit v.5.0.9 [61].

Independent cDNA haplotypes were identified using dnasp v.4.00 [62] and exported in rgl format. A haplotype network was constructed in network (Fluxus Technology, Ltd.) using the median-joining option and redrawn using indesign (Adobe, Inc.). A high level of divergence between two sets of alleles revealed by the haplotype network was the basis for identifying two Types of *ATMYC1* alleles; these Types (I and II) are referenced in other sections.

### Molecular evolution of the *ATMYC1* locus

To examine nucleotide diversity and molecular evolution of the *ATMYC1* locus, the sequence analysis software dnasp v.4.00 [62] was used. The common nucleotide diversity indices, π [63] and θ_w_ [64], were measured across the entire genomic alignment for all sequences and independently for Type I and II data sets. To assess intra-gene variation for nucleotide diversity, a sliding window analysis was run along the full-length (start to stop) cDNA sequence alignment of the 72 *A. thaliana* alleles; window length was set at 45 bp and moved along the alignment at 3 bp intervals.

Because of initial observations of high levels of diversity and divergence between two apparent Types of *ATMYC1* alleles, we tested the null hypothesis of neutral molecular evolution at this locus by measuring the nonsynonymous substitution rate (*K*_A_) and the synonymous substitution rate (*K*_S_). By examining the ratio of *K*_A_/*K*_S_, one may identify signals indicative of positive or purifying selection [50]. *K*_A_/*K*_S_ ratios near one are thought to indicate a neutrally evolving gene or region of a gene, values <<1 are expected to be under purifying selection, and values >>1 indicate positive selection. Because different regions of a gene may experience different forms of selection, a sliding window analysis was used to examine sequence divergence (*K*_A_/*K*_S_) between Types I and II *ATMYC1* alleles; the window size was set at 45 bp, and was moved in 3 bp increments along the length of aligned (inferred) cDNA sequences. These ratio plots were generated in two ways: (1) using local *K*_A_ over local *K*_S_ measures and (2) using local *K*_A_ values over the gene-wide *K*_S_ value. While the former method is the convention, the latter has been suggested as an alternative to deal with false or misleading positives caused by very low local *K*_S_ values [65]. For each window of sequence the *K*_A_/*K*_S_ ratio was calculated using both methods and the results were plotted using sigmaplot (Systat Software, Inc.).

### Testing for an *ATMYC1* Types difference for trichome density

The finding of two highly diverged allele types at the *ATMYC1* locus prompted an investigation of the potential effect of this sequence divergence on trichome density. To this end, trichome density was scored on fifth true leaves for a set of 96 ecotypes of *A. thaliana* (details on this set of ecotypes can be found in [48]) following the methods of Symonds *et al.* [40]. The ecotypes were screened for *ATMYC1* Type using a PCR scheme with Type-specific forward and reverse primers that terminate on multiple sites that are polymorphic between the two Types; that is, only one set of primers yields a product for each ecotype, thus distinguishing the two Types. The trichome data were partitioned into the two allele classes and because the data showed a bimodal distribution, a Kruskal-Wallis rank sum test was performed using mapqtl [66] to test for a significant difference in trichome density between the two groups. Although association mapping in *A. thaliana* ecotype collections is potentially confounded by false positives due to population structure [67], [68], we didn't subsequently correct for population structure given our initial negative result.

### Conservation and placement of L*er*/Col polymorphisms

Outside of the variation that distinguishes the two *ATMYC1* Types (Col and L*er* possess alternative Types), four amino acid replacements differentiate the Col and L*er* alleles. To assess the conservation of these four positions, the Col ATMYC1 protein sequence was submitted to a protein BLAST search and the top 100 hits were aligned and conservation at each of the four sites that differ between the Col and L*er* alleles was evaluated in this alignment.

### Yeast two-hybrid tests for functional differences

Based on the placement and conservation of polymorphisms between the Col and L*er* alleles, two amino acid positions were selected to test for interaction effects with known partners, TTG1 and GL1: A13T and P189A (Col:aa position:L*er*). *ATMYC1* cDNAs were cloned by Reverse Transcription and PCR amplified using start to stop gateway primers and recombined into pDONR/Zeo (Invitrogen). These clones were then modified using Stratagene's QuikChange XL Site-Directed Mutagenesis Kit as recommended by the manufacturer. The Col cDNA was modified to make a version with a T13A change, one with a P189A change and one with both changes. The L*er* cDNA was modified to make a clone with an A13T change, one with an A189P change and one with both. Each of these clones was then recombined into the yeast two-hybrid DNA binding vector pGBT9 RFB. The WDAD (TTG1A) and SRV6 (pGL1A) activation domain vectors were described previously [44]. All clones were sequenced in their entirety. The ATMYC1 yeast vectors were transformed into the Y190 yeast strain. WDAD and SRV6 were then transformed into each of the ATMYC1 yeast lines. The yeast two-hybrid assay was performed as previously described [44] using X-gal as a substrate for β-galactosidase activity and growth on histidine dropout media as interaction markers.

## Supporting Information

Figure S1 First leaf trichome number data for Col-0 and three independent TDNA insertion lines of *ATMYC1*. The mean (+SE) for trichome number on first true leaves is shown for the Col-0 ecotype (n = 20) and three knock-out lines of *ATMYC1*: *atm−1* = SALK_057388 (n = 35), *atm−2* = SALK_056899C (n = 30), *atm−3* = SAIL_227_H01 (n = 35). ANOVA results (*F* (3, 116) = 94.315, *p*<0.001 ) revealed there to be significant differences among genotypes and Tukey-Kramer post-hoc tests showed that each of the three knock-out lines have significantly different trichome number counts than Col-0, but are not significantly different from one another; bars with different letters indicate significantly different samples (*p*<0.01).
(TIF)

Figure S2 Amino acid alignment of the highly variable region of exon six of *ATMYC1* alleles from 72 *A. thaliana* accessions. Accession numbers or names are at left. Types I and II alleles are shown above and below the black horizontal line, respectively. Dots represent amino acids identical to the reference, Col-0 sequence and the amino acid position is indicated across the top. Replacements, relative to Col-0, are shown and the positions of the two outgroup (OG) indels are indicated with arrows.
(TIF)

Table S1 *Arabidopsis thaliana* accessions sequenced for *ATMYC1*. Shown are the 72 *A. thaliana* accessions for which *ATMYC1* was sequenced, with Accession # (ABRC stock #), Accession name, and country of origin. The four accessions that possess the 189A allele are indicated with an *.
(DOC)

## References

[1] Falconer DS, Mackay TFC (1996). Introduction to Quantitative Genetics..

[2] Lynch M, Walsh B (1998). Genetics and Analysis of Quantitative Traits..

[3] Koornneef M, Alonso-Blanco C, Vreugdenhil D (2004). Naturally occurring genetic variation in *Arabidopsis thaliana*.. Annu Rev Plant Biol.

[4] Mackay TF (2010). Mutations and quantitative genetic variation: lessons from *Drosophila*.. Philos Trans R Soc Lond B Biol Sci.

[5] Stinchcombe JR, Hoekstra HE (2008). Combining population genomics and quantitative genetics: finding the genes underlying ecologically important traits.. Heredity.

[6] Atwell S, Huang YS, Vilhjalmsson BJ, Willems G, Horton M (2010). Genome-wide association study of 107 phenotypes in *Arabidopsis thaliana* inbred lines.. Nature.

[7] Bangham J, Obbard DJ, Kim KW, Haddrill PR, Jiggins FM (2007). The age and evolution of an antiviral resistance mutation in *Drosophila melanogaster*.. Proc Biol Sci.

[8] Ehrenreich IM, Torabi N, Jia Y, Kent J, Martis S Dissection of genetically complex traits with extremely large pools of yeast segregants.. Nature.

[9] Weigel D, Nordborg M (2005). Natural variation in *Arabidopsis*. How do we find the causal genes?. Plant Physiol.

[10] El-Assal SED, Alonso-Blanco C, Peeters AJ, Raz V, Koornneef M (2001). A QTL for flowering time in *Arabidopsis* reveals a novel allele of *CRY2*.. Nat Genet.

[11] Flowers JM, Hanzawa Y, Hall MC, Moore RC, Purugganan MD (2009). Population genomics of the *Arabidopsis thaliana* flowering time gene network.. Mol Biol Evol.

[12] Johanson U, West J, Lister C, Michaels S, Amasino R (2000). Molecular analysis of FRIGIDA, a major determinant of natural variation in *Arabidopsis* flowering time.. Science.

[13] Michaels SD, He Y, Scortecci KC, Amasino RM (2003). Attenuation of FLOWERING LOCUS C activity as a mechanism for the evolution of summer-annual flowering behavior in *Arabidopsis*.. Proc Natl Acad Sci USA.

[14] Burke AC, Nelson CE, Morgan BA, Tabin C (1995). Hox genes and the evolution of vertebrate axial morphology.. Development.

[15] Des Marais DL, Rausher MD (2010). Parallel evolution at multiple levels in the origin of hummingbird pollinated flowers in *Ipomoea*.. Evolution.

[16] Jeong S, Rebeiz M, Andolfatto P, Werner T, True J (2008). The evolution of gene regulation underlies a morphological difference between two *Drosophila* sister species.. Cell.

[17] Orr HA, Masly JP, Presgraves DC (2004). Speciation genes.. Curr Opin Genet Dev.

[18] Phadnis N, Orr HA (2009). A single gene causes both male sterility and segregation distortion in *Drosophila* hybrids.. Science.

[19] King MC, Wilson AC (1975). Evolution at two levels in humans and chimpanzees.. Science.

[20] Stern DL, Orgogozo V (2008). The loci of evolution: how predictable is genetic evolution?. Evolution.

[21] Alonso-Blanco C, Aarts MG, Bentsink L, Keurentjes JJ, Reymond M (2009). What has natural variation taught us about plant development, physiology, and adaptation?. Plant Cell.

[22] Rhee SY, Beavis W, Berardini TZ, Chen G, Dixon D (2003). The *Arabidopsis* Information Resource (TAIR): a model organism database providing a centralized, curated gateway to *Arabidopsis* biology, research materials and community.. Nucleic Acids Res.

[23] Tonsor SJ, Alonso-Blanco C, Koornneef M (2005). Gene function beyond the single trait: natural variation, gene effects, and evolutionary ecology in *Arabidopsis thaliana*.. Plant, Cell and Environment.

[24] Singh RS (2003). Darwin to DNA, molecules to morphology: the end of classical population genetics and the road ahead.. Genome.

[25] Theokritoff G (2991). Review: Biotic and abiotic factors in evolution.. BioScience.

[26] Buckley TN (2005). The control of stomata by water balance.. New Phytol.

[27] Tabaeizadeh Z (1998). Drought-induced responses in plant cells.. Int Rev Cytol.

[28] Gilroy S, Jones DL (2000). Through form to function: root hair development and nutrient uptake.. Trends Plant Sci.

[29] Schnall JA, Quatrano RS (1992). Abscisic acid elicits the water-stress response in root hairs of *Arabidopsis thaliana*.. Plant Physiol.

[30] Clauss MJ, Dietel S, Schubert G, Mitchell-Olds T (2006). Glucosinolate and trichome defenses in a natural *Arabidopsis lyrata* population.. J Chem Ecol.

[31] Lopez AV, Vogel S, Machado IC (2002). Secretory trichomes, a substitutive floral nectar source in *Lundia* A. DC. (Bignoniaceae), a genus lacking a functional disc.. Annals of Botany.

[32] Mauricio R (1998). Costs of resistance to natural enemies in field populations of the annual plant *Arabidopsis thaliana*.. Am Nat.

[33] Mendez M, Jones DG, Manetas Y (1999). Enhanced UV-B radiation under field conditions increases anthocyanin and reduces the risk of photoinhibition but does not affect growth in the carnivorous plant *Pinguicula vulgaris*.. New Phytologist.

[34] Saito N, Harborne JB (1992). Correlations betwen anthocyanin type, pollinator and flower color in the Labiatae.. Phytochemistry.

[35] Western TL, Skinner DJ, Haughn GW (2000). Differentiation of mucilage secretory cells of the *Arabidopsis* seed coat.. Plant Physiol.

[36] Ramsay NA, Glover BJ (2005). MYB-bHLH-WD40 protein complex and the evolution of cellular diversity.. Trends Plant Sci.

[37] Hilscher J, Schlotterer C, Hauser MT (2009). A single amino acid replacement in ETC2 shapes trichome patterning in natural *Arabidopsis* populations.. Curr Biol.

[38] Larkin JC, Young N, Prigge M, Marks MD (1996). The control of trichome spacing and number in *Arabidopsis*.. Development.

[39] Mauricio R (2005). Ontogenetics of QTL: the genetic architecture of trichome density over time in *Arabidopsis thaliana*.. Genetica.

[40] Symonds VV, Godoy AV, Alconada T, Botto JF, Juenger TE (2005). Mapping quantitative trait loci in multiple populations of *Arabidopsis thaliana* identifies natural allelic variation for trichome density.. Genetics.

[41] Heim MA, Jakoby M, Werber M, Martin C, Weisshaar B (2003). The basic helix-loop-helix transcription factor family in plants: a genome-wide study of protein structure and functional diversity.. Mol Biol Evol.

[42] Toledo-Ortiz G, Huq E, Quail PH (2003). The Arabidopsis basic/helix-loop-helix transcription factor family.. Plant Cell.

[43] Maes L, Inze D, Goossens A (2008). Functional specialization of the TRANSPARENT TESTA GLABRA1 network allows differential hormonal control of laminal and marginal trichome initiation in *Arabidopsis* rosette leaves.. Plant Physiol.

[44] Payne CT, Zhang F, Lloyd AM (2000). GL3 encodes a bHLH protein that regulates trichome development in *Arabidopsis* through interaction with GL1 and TTG1.. Genetics.

[45] Zhang F, Gonzalez A, Zhao M, Payne CT, Lloyd A (2003). A network of redundant bHLH proteins functions in all TTG1-dependent pathways of *Arabidopsis*.. Development.

[46] Urao T, Yamaguchi-Shinozaki K, Mitsukawa N, Shibata D, Shinozaki K (1996). Molecular cloning and characterization of a gene that encodes a MYC-related protein in *Arabidopsis*.. Plant Mol Biol.

[47] Zimmermann IM, Heim MA, Weisshaar B, Uhrig JF (2004). Comprehensive identification of *Arabidopsis thaliana* MYB transcription factors interacting with R/B-like BHLH proteins.. Plant J.

[48] Nordborg M, Hu TT, Ishino Y, Jhaveri J, Toomajian C (2005). The pattern of polymorphism in *Arabidopsis thaliana*.. PLoS Biol.

[49] Schmid KJ, Ramos-Onsins S, Ringys-Beckstein H, Weisshaar B, Mitchell-Olds T (2005). A multilocus sequence survey in *Arabidopsis thaliana* reveals a genome-wide departure from a neutral model of DNA sequence polymorphism.. Genetics.

[50] Kimura M (1977). Preponderance of synonymous changes as evidence for the neutral theory of molecular evolution.. Nature.

[51] Presgraves DC, Balagopalan L, Abmayr SM, Orr HA (2003). Adaptive evolution drives divergence of a hybrid inviability gene between two species of *Drosophila*.. Nature.

[52] Morohashi K, Grotewold E (2009). A systems approach reveals regulatory circuitry for *Arabidopsis* trichome initiation by the GL3 and GL1 selectors.. PLoS Genet.

[53] Innan H, Tajima F, Terauchi R, Miyashita NT (1996). Intragenic recombination in the Adh locus of the wild plant *Arabidopsis thaliana*.. Genetics.

[54] Kawabe A, Innan H, Terauchi R, Miyashita NT (1997). Nucleotide polymorphism in the acidic chitinase locus (ChiA) region of the wild plant *Arabidopsis thaliana*.. Mol Biol Evol.

[55] Tian D, Araki H, Stahl E, Bergelson J, Kreitman M (2002). Signature of balancing selection in *Arabidopsis*.. Proc Natl Acad Sci U S A.

[56] Hichri I, Heppel SC, Pillet J, Leon C, Czemmel S (2010). The Basic Helix-Loop-Helix Transcription Factor MYC1 Is Involved in the Regulation of the Flavonoid Biosynthesis Pathway in Grapevine.. Mol Plant.

[57] Rockman MV, Stern DL (2008). Tinker where the tinkering's good.. Trends Genet.

[58] Sokal RR, Rohlf FJ (1995). Biometry..

[59] Doyle J, Doyle J (1987). A rapid DNA isolation procedure for small quantitites of fresh leaf material.. Phytochem Bull.

[60] Thompson JD, Gibson TJ, Plewniak F, Jeanmougin F, Higgins DG (1997). The CLUSTAL_X windows interface: flexible strategies for multiple sequence alignment aided by quality analysis tools.. Nucleic Acids Res.

[61] Hall TA (1999). BioEdit: a user-friendly biological sequence alignment editor and analysis program for Windows 95/98/NT.. Nucl Acids Symp Set.

[62] Rozas J, Sanchez-DelBarrio JC, Messeguer X, Rozas R (2003). DnaSP, DNA polymorphism analyses by the coalescent and other methods.. Bioinformatics.

[63] Nei M (1987). Molecular evolutionary genetics..

[64] Watterson GA (1975). On the number of segregating sites in genetical models without recombination.. Theor Popul Biol.

[65] Shiu SH, Karlowski WM, Pan R, Tzeng YH, Mayer KF (2004). Comparative analysis of the receptor-like kinase family in Arabidopsis and rice.. Plant Cell.

[66] Van Ooijen J (2004). MapQTL 5, Software for the mapping of quantitative trait loci in experimental populations..

[67] Aranzana MJ, Kim S, Zhao K, Bakker E, Horton M (2005). Genome-wide association mapping in *Arabidopsis* identifies previously known flowering time and pathogen resistance genes.. PLoS Genet.

[68] Lander ES, Schork NJ (1994). Genetic dissection of complex traits.. Science.

