# Supplementary figures and images for: Natural Allelic Variation Defines a Role for ATMYC1: Trichome Cell Fate Determination
Source: PLoS Genet. 2011 Jun 9;7(6):e1002069. doi: 10.1371/journal.pgen.1002069 (PMC3111535; doi:10.1371/journal.pgen.1002069)

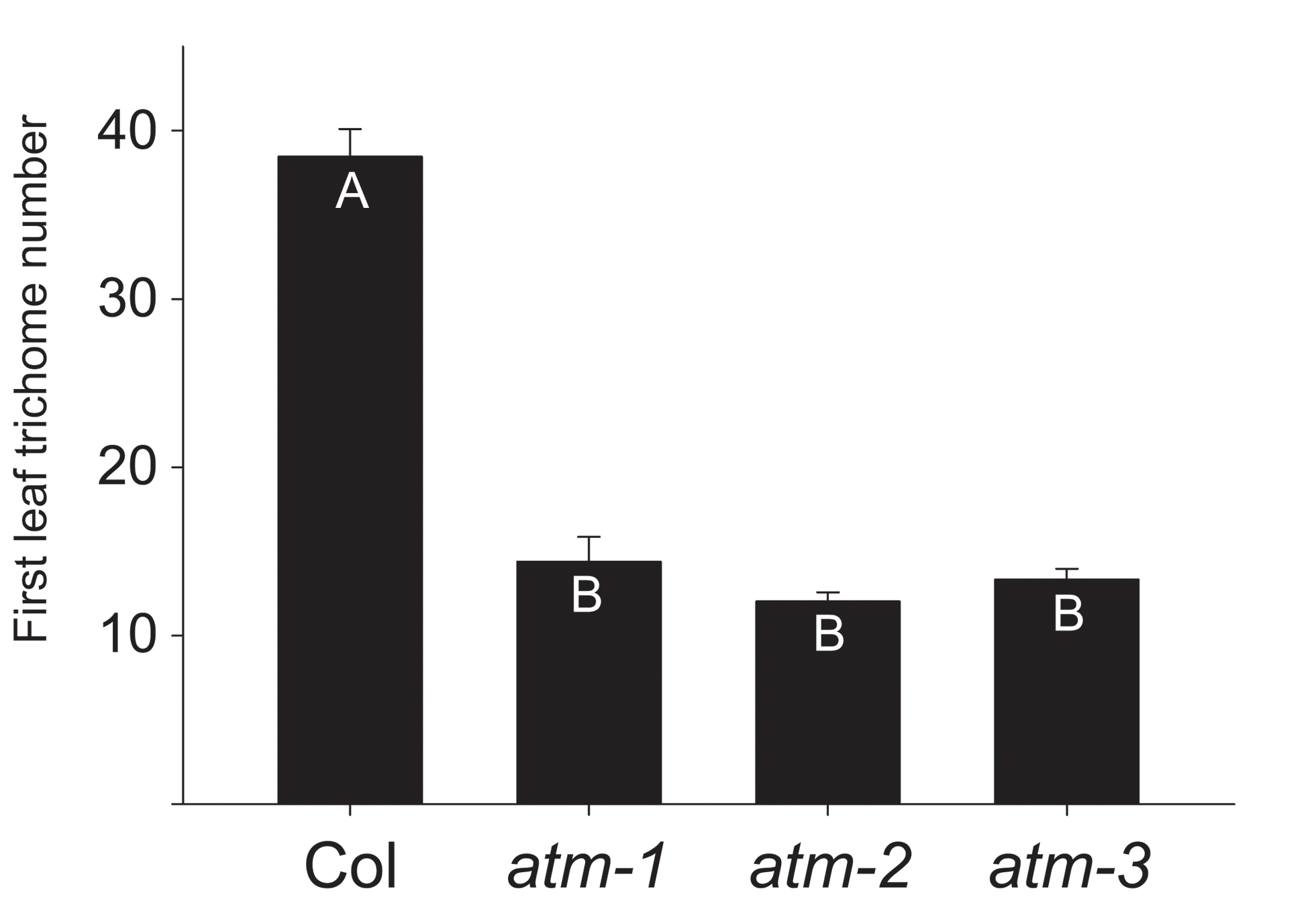

Supplement: Figure S1 — First leaf trichome number data for Col-0 and three independent TDNA insertion lines of ATMYC1. The mean (+SE) for trichome number on first true leaves is shown for the Col-0 ecotype (n = 20) and three knock-out lines of ATMYC1: atm−1 = SALK_057388 (n = 35), atm−2 = SALK_056899C (n = 30), atm−3 = SAIL_227_H01 (n = 35). ANOVA results (F (3, 116) = 94.315, p<0.001 ) revealed there to be significant differences among genotypes and Tukey-Kramer post-hoc tests showed that each of the three knock-out lines have significantly different trichome number counts than Col-0, but are not significantly different from one another; bars with different letters indicate significantly different samples (p<0.01). (TIF) [file pgen.1002069.s001.tif]

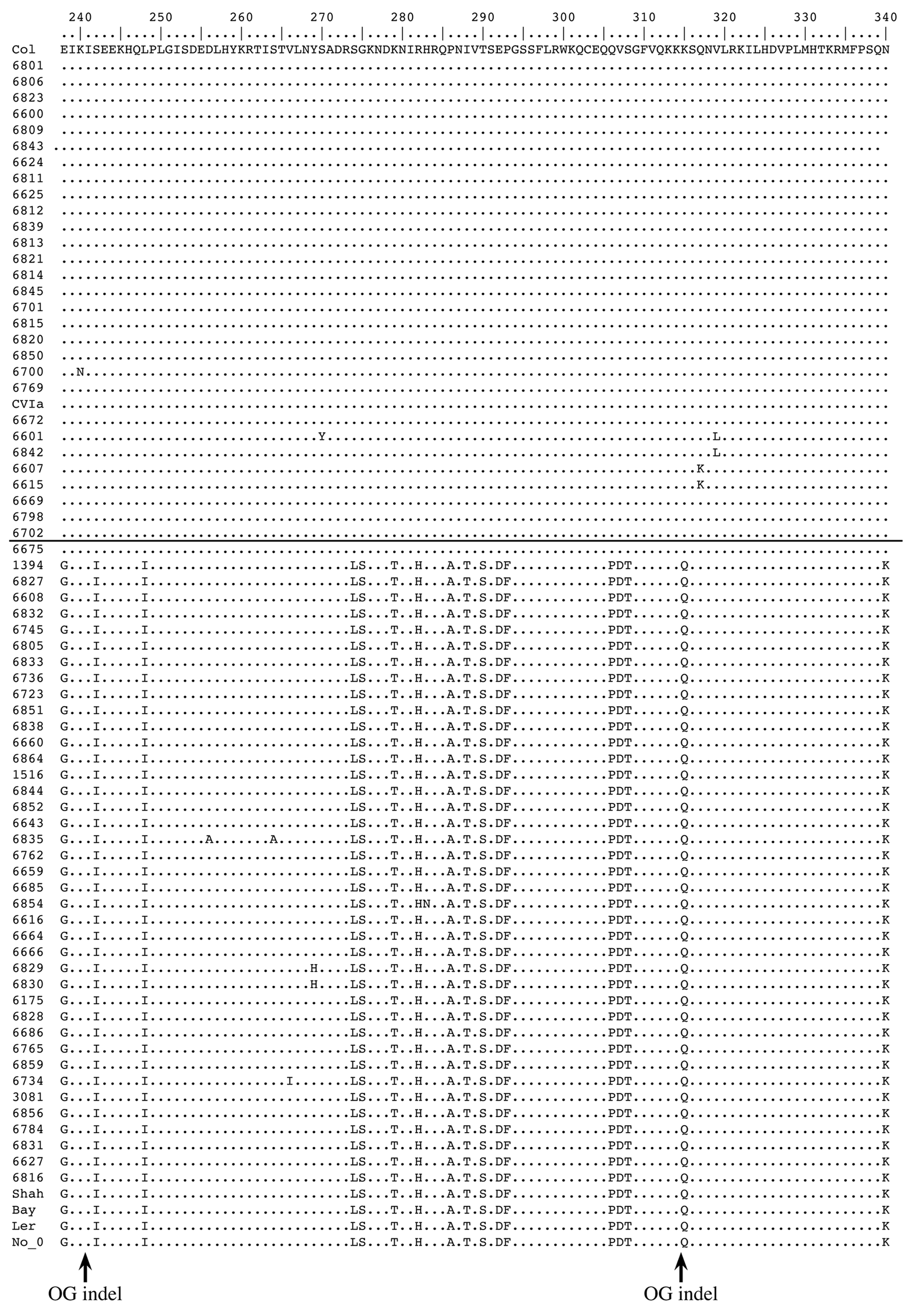

Supplement: Figure S2 — Amino acid alignment of the highly variable region of exon six of ATMYC1 alleles from 72 A. thaliana accessions. Accession numbers or names are at left. Types I and II alleles are shown above and below the black horizontal line, respectively. Dots represent amino acids identical to the reference, Col-0 sequence and the amino acid position is indicated across the top. Replacements, relative to Col-0, are shown and the positions of the two outgroup (OG) indels are indicated with arrows. (TIF) [file pgen.1002069.s002.tif]
