# Supplementary material for: Natural Allelic Variation Defines a Role for ATMYC1: Trichome Cell Fate Determination
Source: PLoS Genet. 2011 Jun 9;7(6):e1002069. doi: 10.1371/journal.pgen.1002069 (PMC3111535; doi:10.1371/journal.pgen.1002069)
Supplement: Table S1 — Arabidopsis thaliana accessions sequenced for ATMYC1. Shown are the 72 A. thaliana accessions for which ATMYC1 was sequenced, with Accession # (ABRC stock #), Accession name, and country of origin. The four accessions that possess the 189A allele are indicated with an *. (DOC) [file pgen.1002069.s003.doc]

| **Supplementary Table 1. *A. thaliana* ecotypes sequenced for *ATMYC1*** | | | | | | |
| --- | --- | --- | --- | --- | --- | --- |
| Accession # | Accession name | Country | Accession # | Accession name | Country |  |
| 1394 | No-0 | Germany | 6805 | No-0 | Germany |  |
| 1516 | Sf-2 | Spain | 6806 | Np-0 | Germany |  |
| 3081 | No-0 | Germany | 6809 | Nok-2 | Netherlands |  |
| 6175 | Kondara | Tadjikistan | 6811 | Nw-0 | Germany |  |
| 6600 | Aa-0 | Germany | 6812 | Nw-1 | Germany |  |
| 6601 | Ag-0 | France | 6813 | Nw-2 | Germany |  |
| 6607 | Ba-1 | U.K. | 6814 | Nw-3 | Germany |  |
| 6608 | Bay-0 | Germany | 6815 | Nw-4 | Germany |  |
| 6615 | Bl-1 | Italy | 6816 | Ob-0 | Germany |  |
| 6616 | Bla-1 | Spain | 6820 | Old-1 | Germany |  |
| 6624 | Bla-12 | Spain | 6821 | Old-2 | Germany |  |
| 6625 | Bla-14 | Spain | 6823 | Ove-0 | Germany |  |
| 6627 | Bs-1 | Switzerland | 6827 | Pa-3 | Italy |  |
| 6643 | Bir-0 | Ireland | 6828 | Per-1 | Russia |  |
| 6659 | Cal-0 | U.K. | 6829 | Per-2 | Russia |  |
| 6660 | Can-0 | Spain | 6830 | Per-3 | Russia |  |
| 6664 | Chi-0 | Russia | 6831 | Pf-0 | Germany |  |
| 6666 | Chi-2 | Russia | 6832 | Pi-0 | Austria |  |
| 6669 | Co-1 | Portugal | 6833 | Pi-2 | Austria |  |
| 6672 | Co-4 | Portugal | 6835 | Pla-1 | Spain |  |
| 6675 | Cvi-0 | Cape Verdi | 6838 | Pn-0 | France |  |
| 6685 | Dra-0 | Czech Rep. | 6839 | Po-0 | Germany |  |
| 6686 | Dra-1* | Czech Rep. | 6842 | Pog-0 | Canada |  |
| 6700 | Est-0 | Russia | 6843 | Pt-0 | Germany |  |
| 6701 | Est-1 | Russia | 6844 | Ra-0 | France |  |
| 6702 | Et-0 | France | 6845 | Rd-0 | Germany |  |
| 6723 | Gr-1 | Austria | 6850 | Rsch-4 | Russia |  |
| 6734 | Hau-0 | Denmark | 6851 | Ru-0 | Germany |  |
| 6736 | Hi-0 | Netherlands | 6852 | Se-0 | Spain |  |
| 6745 | Jl-3 | Czech Rep. | 6854 | Sap-0 | Czech Rep. |  |
| 6762 | Kn-0 | Lithuania | 6856 | Sav-0 | Czech Rep. |  |
| 6765 | La-0* | Poland | 6859 | Sg-2* | Germany |  |
| 6769 | Lc-0 | U.K. | 6864 | Ste-0 | Germany |  |
| 6784 | Lm-2 | France | Col | Col | USA |  |
| 6798 | Mir-0 | Italy | L*er* | L*er** | Poland |  |
| 6801 | Na-1 | France | 57924 | Sha | Tajikistan |  |

* identifies accessions that carry the 189A allele.
